# A Randomized Clinical Trial Comparing Three Different Exercise Strategies for Optimizing Aerobic Capacity and Skeletal Muscle Performance in Older Adults: Protocol for the DART Study

**DOI:** 10.3389/fmed.2019.00236

**Published:** 2019-10-22

**Authors:** Dallin Tavoian, David W. Russ, Timothy D. Law, Janet E. Simon, Paul J. Chase, Emily Hill Guseman, Brian C. Clark

**Affiliations:** ^1^Ohio Musculoskeletal and Neurological Institute, Ohio University, Athens, OH, United States; ^2^Laboratory for Integrative Muscle Biology, Division of Physical Therapy, Ohio University, Athens, OH, United States; ^3^Division of Athletic Training, School of Applied Health Sciences and Wellness, Ohio University, Athens, OH, United States; ^4^Division of Exercise Physiology, School of Applied Health Sciences and Wellness, Ohio University, Athens, OH, United States; ^5^Diabetes Institute, Ohio University, Athens, OH, United States; ^6^Department of Family Medicine, Heritage College of Osteopathic Medicine, Ohio University, Athens, OH, United States; ^7^Department of Biomedical Sciences, Ohio University, Athens, OH, United States

**Keywords:** aging, exercise, intervals, resistance, aerobic, power, VO_2_, muscle

## Abstract

**Background:** Age-related declines in physical function lead to decreased independence and higher healthcare costs. Individuals who meet the endurance and resistance exercise recommendations can improve their physical function and overall fitness, even into their ninth decade. However, most older adults do not exercise regularly, and the majority of those who do only perform one type of exercise, and in doing so are not getting the benefits of endurance or resistance exercise. Herein we present the study protocol for a randomized clinical trial that will investigate the potential for high-intensity interval training (HIIT) to improve maximal oxygen consumption, muscular power, and muscle volume (primary outcomes), as well as body composition, 6-min walk distance, and muscular strength and endurance (secondary outcomes).

**Methods and Analysis:** This is a single-site, single-blinded, randomized clinical trial. A minimum of 24 and maximum of 30 subjects aged 60–75 that are generally healthy but insufficiently active will be randomized. After completion of baseline assessments, participants will be randomized in a 1:1:1 ratio to participate in one of three 12-week exercise programs: stationary bicycle HIIT, stationary bicycle moderate-intensity continuous training (MICT), or resistance training. Repeat assessments will be taken immediately post intervention.

**Discussion:** This study will examine the potential for stationary bicycle HIIT to result in both cardiorespiratory and muscular adaptations in older adults. The results will provide important insights into the effectiveness of interval training, and potentially support a shift from volume-driven to intensity-driven exercise strategies for older adults.

**Clinical Trial Registration:** This trial is registered with ClinicalTrials.gov (registration number: NCT03978572, date of registration June 7, 2019).

## Introduction

Nearly half of US adults over age 60 report difficulty performing one or more activities of daily living essential to maintaining independence, a fraction that has remained stable over the last 20 years despite an overall increase in the mean age of the US population (1). Increasing numbers of older adults with disabilities will continue to drive up healthcare costs, making maintenance of health and independence a top priority for both middle-aged and older adults (2). Contributors to this functional decline include poor cardiorespiratory fitness and skeletal muscle impairments (3–5), which can partially be attributed to reduced physical activity with age (6–8). Exercise is effective at maintaining function in older adults, as evidenced by data indicating those who take part in supervised exercise programs demonstrate improvements in functional outcomes (9–11). The fact that adaptations have been seen in the oldest adults (90+ years) is particularly encouraging (12–14).

Unfortunately, <12% of older adults meet the exercise recommendations provided by the American College of Sports Medicine (ACSM) (1, 15). These recommendations include weekly accumulation of at least 150 min of moderate intensity aerobic activity or 75 min of vigorous aerobic (endurance) activity, and at least 2 days of resistance exercise to improve muscular endurance, power, and strength (15). In 2014, 36.5% of adults over the age of 65 met the aerobic exercise recommendations, while only 16.5% met the resistance exercise recommendations; only 11.7% met both aerobic and resistance recommendations simultaneously (1). Consequently, the majority of older adults who do exercise are either not getting the muscular adaptations necessary for maintaining independence, or they are not getting the necessary cardiorespiratory adaptations. However, certain types of exercise may be able to induce both cardiorespiratory and muscular adaptations in older adults.

Resistance exercise appears to have minor effects on cardiovascular disease (CVD) risk factors, though its benefits include the prevention of musculoskeletal injuries, muscle wasting, and impairments in physical function (16). On the other hand, aerobic exercise is effective at reducing CVD risk by improving heart, lung, and metabolic function, though it appears to have little effect on muscular properties (17). However, recent work indicates that the absence of muscular adaptations in response to aerobic training may be related to exercise intensity and mode of exercise (18, 19). The most popular form of physical activity in older adults is walking, and recommendations for older adults promote walking as the primary means to increase physical activity levels (15, 20, 21). While walking may challenge older adults and result in cardiovascular improvements, the relatively low intensity of muscular contractions would be unlikely to elicit hypertrophy or functional adaptations of the leg muscles (22). Even higher intensity running does not seem to result in increased muscle size or strength (19, 23), and running may blunt the muscular benefits of resistance training when performed concurrently (24). In contrast, stationary bicycle training can increase muscular strength and size (25–27), and does not seem to interfere with the muscular adaptations to resistance training (24). Bicycle high-intensity interval training (HIIT) in particular may be the ideal form of aerobic exercise able to elicit muscular adaptations. Young adults performing bicycle HIIT demonstrate improved muscle strength (28) and power (29, 30), along with enhanced cardiorespiratory function (31).

HIIT has gained popularity in recent years, with most research in middle-aged and older cardiac rehabilitation patients (32, 33). Compared to traditional aerobic training, HIIT typically has a greater effect on VO_2_max (9.1% greater increase on average) and other CVD risk factors (e.g., cholesterol, blood pressure) (33). While its success in rehabilitation is encouraging, the effectiveness of HIIT as a general exercise strategy for older adults has not been adequately investigated, with few studies reporting physiological adaptations to HIIT in older adults (33–44). Furthermore, limitations make it difficult to generalize these studies to a healthy aging population. Specifically, these studies have had one or more of the following methodological confounds:

1. They lacked an active control group that followed established exercise recommendations.
2. They were short-term interventions.
3. The target population had overt diseases/health conditions, which may limit generalizability to the large number of older adults that are generally healthy.

The lack of investigations that directly compare unique exercise strategies against established strategies represents a critical barrier to progress in the field of healthy aging. With this in mind, the DART Study (Dual-benefits of Aerobic and Resistance Training) is a phase 1B proof-of-concept, proof-of mechanism clinical trial that seeks to determine if bicycle HIIT is a more efficient standalone strategy to improve cardiovascular and lower extremity muscular function than established resistance or aerobic exercise training programs. We will test this “dual-benefits hypothesis” in generally healthy older adults that have yet to develop mobility limitations, but who, without intervention, could very likely develop mobility limitations in the future due to inactivity and normal time course of aging.

## Methods and Analysis

### Design

**Figure 1:**
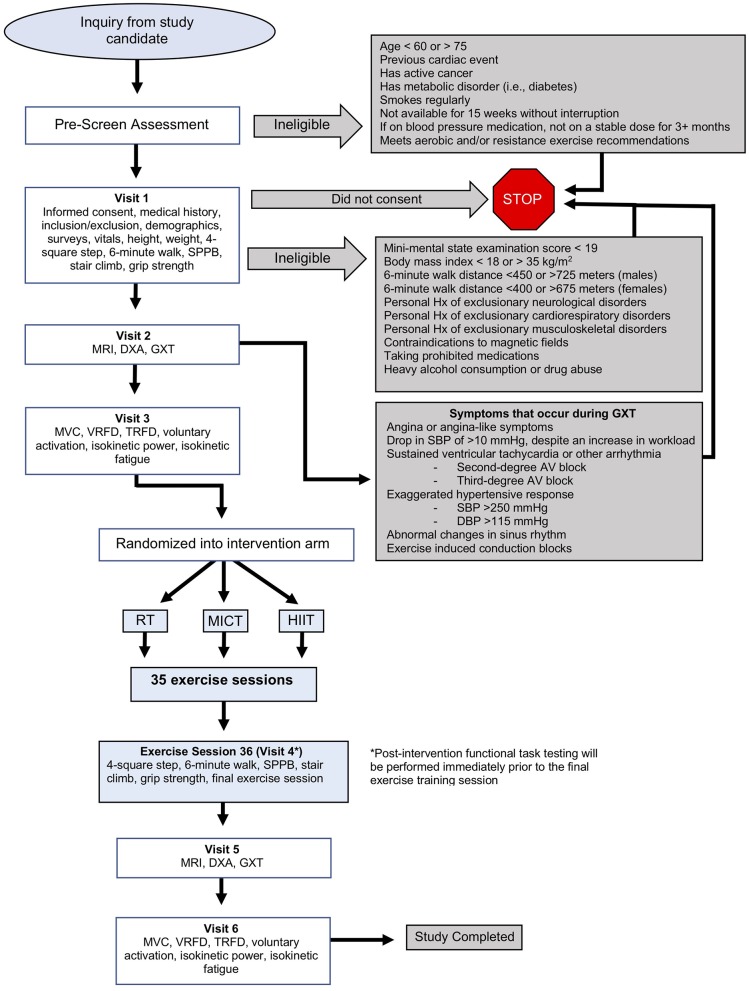
Detailed overview of DART Study protocol. DBP, diastolic blood pressure; DXA, dual-energy X-ray absorptiometry; GXT, graded exercise test; HIIT, high-intensity interval training; Hx, history; MICT, moderate-intensity continuous training; MRI, magnetic resonance imaging; RT, resistance training; SBP, systolic blood pressure; SPPB, short physical performance battery; TRFD, twitch-evoked rate of force development; VRFD, voluntary rate of force development.

This is a single-blinded (outcomes assessor), single-site, randomized control trial. It is a three (group) by two (time) repeated measures factorial design. The overall study is illustrated in Figure 1.

### Study Subjects

A minimum of 24 (and up to 30) subjects aged 60–75 years of age that are generally healthy but sedentary will be recruited, enrolled, and randomized in this study (*n* = 8–10 per group). Potential subjects will be recruited from the local community by way of flyers, community events, or emails to individuals who have previously participated in studies with the Ohio Musculoskeletal and Neurological Institute at Ohio University in Athens, Ohio, USA. All interested individuals will complete a pre-screening phone interview, and all that are not ruled ineligible will be invited for in-person screening. Written informed consent will be obtained from each subject in accordance with the Declaration of Helsinki. Ethical Approval for this study has been obtained from the Ohio University Institutional Review Board (protocol number 18-F-55).

Eligible subjects will be insufficiently active. We define “insufficiently active” as not meeting either the endurance or resistance exercise recommendations for older adults, as set forth by the American College of Sports Medicine (ACSM), for three consecutive months. The current ACSM recommendations for endurance training are 150–300 min per week of moderate-intensity activity (perceived exertion of 5–6 on 0–10 scale), or 75–150 min per week of vigorous-intensity activities (perceived exertion of 7–8 on 0–10 scale) (15). The recommendations for resistance training are at least 2 days per week of progressive weight training activities that use the major muscle groups (perceived exertion of 5–8 on 0–10 scale) (15). Additionally, the subjects must not be highly active outside of a structured exercise program (i.e., consistent hard physical labor). They must also have a 6-min walk distance within a normal range for adults aged 60–75. We calculated a normal 6-min walk range by averaging 6-min walk values from multiple studies, plus or minus one standard deviation (45–48). The calculated 6-min walk range for females was 400–675 meters, and for males was 450–725 meters. Table 1 describes inclusion and exclusion criteria in detail. These criteria are designed to recruit a generally healthy, but insufficiently active population, and exclude individuals with poor health and physical function where there could be concerns about a subject's ability to appropriately perform the testing and exercise prescription.

**Table 1:** Inclusion and exclusion criteria.

Inclusion Criteria: •Age 60–75 years with no significant health issues or conditions that, in the investigators' opinion, would limit the subject's ability to complete the study per protocol or that would impact the capability to get an accurate measurement of study endpoints. •Body mass index between 18 and 40 kg/m^2^. •Willingness to maintain current diet and adhere to the intervention programs described for the study and willing to undergo all testing procedures. •Able to read, understand, and complete study-related questionnaires. •Able to read and understand, and willing to sign the informed consent form (ICF). •Six-min walk distance of 450–725 meters for men and 400–675 meters for women.Exclusion Criteria: •Short physical performance batter (SPPB) score <8. •Any activities of daily living disability (difficulty feeding, dressing, continence, bathing, toileting, and transferring). •Lives in a nursing home or assisted living facility. •Known neuromuscular or neurological conditions affecting somatosensory or motor function or control (e.g., hemiplegia, multiple sclerosis, peripheral neuropathy, Parkinson's disease, Myasthenia Gravis, Ataxia, Apraxia, post-polio syndrome, mitochondrial myopathy, etc.). •Unable to communicate because of severe hearing loss or speech disorder. •Severe visual impairment, which would preclude completion of the assessments. •Cancer requiring treatment currently or in the past 2 years (except primary non-melanoma skin cancer or *in situ* cervical cancer). •Hospitalization (medical confinement for 24 h), or immobilization, or major surgical procedure requiring general anesthesia within 12 weeks prior to screening, or any planned surgical procedures during the study period. •Chronic or relapsing/remitting gastrointestinal disorders such as inflammatory bowel disease and irritable bowel syndrome. •Known history of human immunodeficiency virus (HIV) antibody at screening. •Use of systemic glucocorticoids. •Any history of angina pectoris. •Any history of heart failure. •Any history of myocardial infarction. •Any coronary artery bypass graft or percutaneous coronary intervention. •Heart disease that limits exercise (valvular, congenital, ischemic, and hypertrophic cardiomyopathy). •Complex ventricular arrhythmias or heart block. •Chronic obstructive pulmonary disease, cerebrovascular disease, or peripheral vascular disease. •Diabetes mellitus •Severe neuropathy. •Mini-mental state exam score below 19. •Psychiatric conditions that warrant acute or chronic therapeutic intervention (e.g., major depressive disorder, bipolar disorder, panic disorder, schizophrenia) that in the investigators' opinion may interfere with the conduct of study procedures. •Unable to undergo magnetic resonance imaging (MRI) (e. g., body containing any metallic medical devices or equipment, including heart pacemakers, metal prostheses, implants or surgical clips, any prior injury from shrapnel or grinding metal, exposure to metallic dusts, metallic shavings or having tattoos containing metallic dyes). •Unable to reliably undergo exercise or strength tests described for this study. •Participation in progressive resistance exercise 2 or more days/week for most weeks over the 24 weeks prior to screening, OR 150+ min of accumulated aerobic exercise each week for most weeks over the 24 weeks prior to screening. •Current self-reported activity level that, in the investigators' opinion, is considered highly active for older adults. •Participation in any clinical trial within 12 weeks prior to screening. •Limb amputation (except for toes). •Bone fracture within 24 weeks prior to screening. •Any disorder that will not allow completion of the motions required for resistance or aerobic exercise. •Conditions (such as myasthenia gravis, myositis, muscular dystrophy, or myopathy, including drug-induced myopathy) leading to muscle loss, muscle weakness, muscle cramps, or myalgia. •Acute viral or bacterial upper or lower respiratory infection at screening. •Abnormal or uncontrolled blood pressure (BP) at the screening visit defined as BP > 170/100 mmHg. If taking anti-hypertensive medication, have to be on stable doses of medication for more than 3 months. •Current or recent history (within 1 year of screen) of heavy alcohol consumption or drug abuse that in the investigators' opinion may interfere with the conduct of study procedures. •Reports being pregnant, lactating, or that they anticipate becoming pregnant in the next 3-months. If a woman becomes pregnant while on study protocol, they will be withdrawn from the study. Prohibited Medications: Medications that, in the PIs opinion, would confound study integrity by interacting with study outcomes. For instance: •Anti-obesity drugs, nutraceuticals, and dietary supplements that may affect body mass and body composition. •Any drug or supplement known to influence muscle mass or performance including but not limited to anabolic steroids, insulin-like growth factor 1, growth hormone, replacement androgen therapy, anti-androgen therapy.

### Randomization

Subjects will be randomized in a 1:1:1 ratio to receive a 12-week exercise program consisting of either resistance training (RT), moderate-intensity continuous training (MICT) on a stationary bicycle, or HIIT on a stationary bicycle. Due to the small sample size in each arm, permuted-block randomization will be used via computer-generated random numbers to ensure equal sample size. Specifically, we will create blocks of three with each treatment permuted within each block. The study subjects will be enrolled and assigned to their respective interventions by an unblinded project manager.

#### Implementation

The allocation sequence will be generated by a biostatistician. Subjects will be enrolled in the study and assigned to their intervention group by the project manager.

#### Blinding

Due to the nature of the intervention neither subjects nor staff can be blinded to allocation. The outcomes assessor and data analyst will be blinded after study completion by having the subjects' demographic and intervention group information coded.

### Sample Size

In this proof-of-concept, proof-of-mechanism trial, we report sample size estimates based on previous recommended literature (49). A sample size of ~8 subjects per group will detect a moderate effect. Sample size calculation was based on expected effect sizes for the HIIT cycle, MICT cycle, and RT groups for the primary outcomes of V0_2_max and thigh muscle CSA. Consistent with our “dual benefits hypothesis” we assumed, based on the literature, an 8% increase in thigh CSA for both the HIIT and RT groups (50) and 2% increase for the MICT group (51) and common SD across all groups of 4%. With the assumption of a 2-sided test and alpha level of significance equal to 0.05 an *n* = 7/group yields power of 0.83. With respect to VO_2_max we assumed a 32% increase in the HIIT group, a 15% increase in the MICT group, and a 10% increase in the RT group and common SD across all groups of 14% (52). With the assumption of a 2-sided test and alpha level of significance equal to 0.05 an *n* = 8/group yields power of 0.80. In line with other proof-of-concept, proof-of-mechanism trials, no statistical control for type-I error from multiple comparisons will be considered, and *p*-values will be interpreted with care, as descriptive weights of evidence rather than as confirmatory claims. Lastly, this proof of concept, proof of mechanism trial is needed to test the complex interventions proposed, and the effect sizes calculated from this trial could be used to estimate sample size for a future large-scale clinical trial. Accordingly, we plan to enroll an *n* = 8–10 subjects per group.

### Study Timeline

This study will have a screening/baseline assessment period of 21 days (maximum) with three sessions spaced at least 48 h apart, a 12-week exercise training period, and a post-intervention assessment period of 10 days (maximum) with two sessions spaced at least 48 h apart. Subjects will visit Ohio University's Clinical and Translational Research Unit facilities prior to the intervention for baseline assessments. During Visit 1 we will obtain informed consent and conduct a full medical history screening and a short physical performance battery (SPPB) (53) to determine if candidates meet the inclusion/exclusion criteria. Subjects who meet the criteria will be enrolled in the study and complete a series of clinical and physiological outcome measures over the three baseline visits. Upon completion of baseline assessments, subjects will be randomized into one of the three exercise groups for the 12-week exercise intervention. All exercises will be performed on site and supervised by an exercise professional 3 days per week. All baseline assessments will be repeated upon completion of the exercise intervention. A table of events for the study is illustrated in Table 2.

**Table 2:** Schedule of events for all groups.

	**Baseline period**			**Exercise intervention**		**Follow-up**	
	**Visit 1^**c**^**	**Visit 2^**c**^**	**Visit 3^**c**^**	**Sessions 1–35^**a**^**	**Session 36 (visit 4)^**b**^**	**Visit 5^**d**^**	**Visit 6^**d**^**
Day (window)	−21 to −5	−19 to −3	−17 to −1	1 to 84	80 to 83	84 to 92	86 to 94
**SCREENING/BASELINE:**							
Informed consent	X						
Medical history	X						
Inclusion/exclusion	X						
Demographics	X						
Vitals	X						
Height and weight	X						
**SURVEYS:**							
PASE	X						
MMSE	X						
SEE	X						
**EXERCISE SESSIONS:**							
Exercise				X	X		
**FUNCTIONAL TASKS:**							
4SST	X				X		
Six-min walk	X				X		
SPPB	X				X		
Stair climb	X				X		
Grip strength	X				X		
**MEDICAL IMAGING:**							
MRI		X				X	
DXA		X				X	
**CARDIORESPIRATORY:**							
GXT		X				X	
**MUSCULAR TESTING:**							
MVC			X				X
Ballistic			X				X
Twitch force			X				X
VA			X				X
Isokinetic power			X				X
Isokinetic fatigue			X				X
**RANDOMIZATION**			X				

a *Exercise training sessions will be performed three times per week with at least 1 day between sessions, and no more than two exercise sessions on consecutive days in the same week*.

b *Post-intervention functional task testing will be performed immediately prior to the final exercise training session (session 36)*.

c *Baseline testing will be completed within 21 days of Visit 1, with at least 2 days between testing sessions*.

d *Post-intervention testing will be completed within 10 days of the final exercise session, with at least 2 days between testing sessions. 4SST, 4-square step test; DXA, dual-energy X-ray Absorptiometry; GXT, graded exercise test; MMSE, mini-mental state examination; MRI, magnetic resonance imaging; MVC, maximal voluntary contraction; PASE, physical activity scale for the elderly; SEE, self-efficacy for exercise scale; SPPB, short physical performance battery; VA, voluntary activation*.

### Outcome Measures

#### Primary Outcomes

##### Knee extensor isokinetic power

Maximal isokinetic power will be measured from the knee extensors. Peak torque will be recorded from the non-dominant leg using a Biodex System 4 Dynamometer (Biodex Medical Systems, Inc., Shirley, NY). The subject's leg will be immobilized against the lever arm with the distal end of the lever arm secured three inches superior to the medial malleolus. The axis of the lever arm will be centered at the lateral knee joint and knee range of motion will be obtained by having the subject extend their knee as far as possible against the lever arm. The maximal knee extension angle will be recorded, and the knee extension limit will be set 10° less than the maximal knee extension angle. The subject will then relax to allow the leg to return to neutral position and the knee flexion limit will be set at 80° of knee flexion. The speed of the lever arm will be set at 60°/s for both extension and flexion. The subject will then extend the knee with maximal effort until they reach the knee extension limit, and then immediately flex the knee with maximal effort until they reach the knee flexion limit. The time-series torque signal will be collected at 500 Hz by a Biopac MP150 system (Biopac Systems Inc., Santa Barbara, CA, USA). The subject will complete six isokinetic trials, with 30 s rest between trials. The average of the three highest peak torque values for both extension and flexion will be recorded at baseline and post-intervention, and percent change from baseline will be used for analysis.

##### Maximal oxygen uptake (VO_2_max)

VO_2_max will be obtained with a ParvoMedics TrueOne 2400 metabolic measuring system with a Hans Rudolf 3813 (Shawnee Mission, KS, USA) pneumotachometer to measure ventilation. The TrueOne 2400 is a mixing chamber system that uses a paramagnetic oxygen analyzer (range 0–25%) and an infrared, single beam, single wavelength carbon dioxide analyzer (range 0–10%). Prior to each test the system will be allowed to heat up for 30 min, and then will be calibrated according to the manufacturer's recommendations. This consists of a room air auto-calibration and a gas calibration with a single gas tank (16.00% O_2_, 4.008% CO_2_). Additionally, the flow meter will be calibrated with a 3.000-liter Hans Rudolf 5530 series syringe, with a 5-stroke calibration using different flow rates for each stroke. Ten ECG electrodes will be placed on the subject's body according to Mason-Likar procedures (54, 55). Prior to placement, the areas will be shaved with a disposable razor, wiped with an alcohol pad, and then lightly abraded with fine-grit sandpaper. The subject will be fitted with a Hans Rudolf Oro-Nasal reusable facemask (Shawnee Mission, KS, USA) with a 2-way non-rebreathing valve connected to the metabolic cart with large-bore, low-resistance tubing. The testing protocol will be explained to the subject, as well as how to communicate with investigators while wearing the facemask (i.e., hand signals). Resting VO_2_ values will be collected from the subject after 3 min of rest in a seated position on the cycle ergometer. Resting blood pressure will be taken immediately after resting VO_2_ is obtained. The subject will begin cycling on a magnetically braked cycle Lode Corival CPET ergometer (Lode B.V., Groningen, NL) at 60–80 RPMs (depending on subject comfort level) with a starting power output of 15 watts (W) for 1 min, collecting VO_2_ values every 20 s. Power output will be increased by 15 W every minute until subject can no longer continue the test or criteria have been met (Table 3). With 20 s remaining in each stage, the subject will indicate their RPE on a 6–20 Borg scale (56). With 10 s left in each stage, heart rate will be recorded from ECG readouts. Blood pressure will be taken at the beginning of even numbered stages. Subjects will be verbally encouraged throughout the test, and airflow will be provided through the use of a rotating fan. After test termination, the subject will perform a 5-min cycling cool down at 30 W. Blood pressure will be taken every 5 min for 20 min after the completion of the graded exercise test (GXT). Maximal heart rate will be determined from ECG readings during the final stage of the GXT for all subjects. Absolute VO_2_max measured in L/min will be recorded at baseline and post-intervention, and change from baseline will be used for analysis. Additionally, VO_2_max relative to body mass measured in mL/kg/min will be recorded at baseline and post-intervention, and change from baseline will be used for analysis.

**Table 3:** Criteria for test termination during graded exercise testing.

**Criteria for maximal effort-related test termination**	**Criteria for health concern-related test termination**
•Subject requests to stop •Physical or verbal manifestations of severe fatigue •Failure of heart rate to increase with increased exercise intensity •Unable to maintain a cycle frequency of 50 RPMs for >5 sOr 2 of the 4 following criteria:•RPE of 17 or greater (Borg 6–20 scale) •Peak heart rate that is 85% of age-predicted maximal heart rate (220-age) •Plateau of VO2 •Respiratory exchange ratio (RER) of 1.1 or higher	•Angina or angina-like symptoms (subjective score of 2 or greater on ACSM angina scale) •Shortness of breath, wheezing, leg cramps, or claudication (subjective score of 3 or greater on ACSM claudication scale) •Signs of poor perfusion: light-headedness, confusion, ataxia, pallor, cyanosis, nausea, or cold and clammy skin •Drop in systolic blood pressure of >10 mm Hg, despite an increase in workload •Central nervous system symptoms (e.g., ataxia, dizziness, or near syncope) •Sustained ventricular tachycardia or other arrhythmia, including second- or third-degree atrioventricular block, that interferes with normal maintenance of cardiac output during exercise •Exaggerated hypertensive response (systolic blood pressure >250 mm Hg or diastolic blood pressure >115 mm Hg) •Abnormal changes in sinus rhythm •Exercise induced conduction blocks

*ACSM, American College of Sports Medicine; RPE, rating of perceived exertion; RPM, rotations per minute; VO2, volume of oxygen consumption*.

##### Quadriceps muscle volume

Quadriceps muscle volume will be obtained via magnetic resonance imaging (MRI) scans performed with a 0.25-Tesla Musculoskeletal MRI system (Esaote G-Scan Brio, Genoa, Italy) to acquire contiguous transverse T-1 weighted spin echo image slices in the thigh region with a slice thickness of 10 mm and an inter-slice distance of 10 mm. The isocenter will be positioned at mid-thigh, midway between the patella and the inguinal crease, and the subjects will be supine. Images will be transferred to a computer for calculation of quadriceps anatomical cross-sectional area (CSA). Beginning with the slide with the first discernable visual of the rectus femoris and including the subsequent four proximal slides, quadriceps muscle area will be traced using a polygon tool, excluding bone, as well as fat tissue surrounding the muscles (MIPAV version 7.3.0). Intramuscular fat will then be subtracted by applying a shading correction to each slide, determining average voxel density and standard deviation voxel density from a sample of the lightest area of fat tissue, computing a cutoff value at three standard deviations darker than the sample voxel density, and excluding all pixels with a voxel density at or below the computed value. Pre- and post-intervention slides will be displayed simultaneously, and slides will be visually compared to ensure that tracing patterns are identical and that the same structures are excluded (i.e., neurovascular bundle, intermuscular fat) on both slides before CSA values are recorded. This process will be completed for each analyzed slide, resulting in five measures of quadriceps CSA with intermuscular and intramuscular fat excluded for both pre and post time points. Muscle volume will then be calculated using the Cavalieri method [MV = T (A_1_ + A_2_ + A_3_ + A_4_ + A_5_)], where MV = muscle volume, T = the known distance between slices, and A = area (57). Quadriceps muscle volume will be recorded at baseline and post-intervention, and percent change from baseline will be used for analysis.

#### Secondary Outcomes

##### Knee extensor isometric strength

Maximal isometric force production will be measured via three maximal voluntary contractions (MVCs) of the knee extensors while the subject is positioned in the Biodex Dynamometer as described above, with the lever arm immobilized at 90° of knee flexion. The subject will be instructed to gradually increase force for the first second, and then exert maximal effort for ~3–4 s. The subject will perform three MVCs with a 30–60 s rest period between contractions. Verbal encouragement will be provided during each trial. The time-series torque signal will be collected at 500 Hz by a Biopac MP150 system (Biopac Systems Inc., Santa Barbara, CA, USA). The trial with the highest value will be recorded at baseline and post-intervention, and percent change from baseline will be used for analysis.

##### Knee extensor isokinetic fatigue

Fatigue resistance of the knee extensors will be measured with the subject positioned in the Biodex dynamometer as described above. Subjects will be asked to perform a series of isokinetic leg extensions at 120°/s (the flexion component will be passive at a speed of 240°/s). First, study subjects will perform three isokinetic extensions recorded as pre-fatigue peak torque values. Next, study subjects will be given 3 min of rest before beginning the fatigue portion of the test. For the fatigue test, subjects will perform 120 consecutive maximal isokinetic leg extension contractions (test time ~4-min). Peak force for each of the 120 contractions will be summed to calculate total work output and will be recorded at baseline and post-intervention, and change from baseline will be used for analysis. The subject will be verbally encouraged throughout the test. Lastly, post-fatigue peak torque will be assed at 2, 5, and 10 min after the completion of the fatigue test. For all fatigue measurement, the time-series torque signal will be collected at 500 Hz by a Biopac MP150 system (Biopac Systems Inc., Santa Barbara, CA, USA). Average torque of three contractions at each of the post-fatigue timepoints will be recorded and expressed relative to the pre-fatigue torque at baseline and post-intervention, and percent change from baseline will be used for analysis.

##### Six-minute walk distance

A 6-min walk distance test will be performed in a 30-meter hallway marked off with cones at either end, and distance marked every three meters. The subject will start at the end of the hallway (starting cone) and be instructed to walk as quickly as they can for 6 min. They will walk toward the end of the hallway, around the second cone, and then back toward the starting cone. The subject will complete as many laps as possible within 6 min, rounding the cones each lap. Subjects will be given feedback on elapsed/remaining time every 30 s, and encouraged to continue walking as quickly as possible. Distance covered in 6 min will be recorded to the nearest meter at baseline and post-intervention, and change from baseline will be used for analysis.

##### Body composition

Total body fat mass will be obtained via whole-body dual-energy X-ray absorptiometry (DXA) scans (Hologic Discovery QDR model Series, Waltham, MA, USA) using the system's software package (Hologic APEX, Version 4.0.2). Subjects will be scanned at the same time of day pre- and post-intervention and will be encouraged to maintain a similar sleeping and eating schedule for both scans. Subjects will be advised to report to the laboratory in a hydrated state and will be given scrubs to wear during the scan. They will also be given the opportunity to use the restroom prior to the scan. Care will be taken to follow The International Society for Clinical Densitometry guidelines for positioning during the scan (58). Total body fat mass will be recorded at baseline and post-intervention, and percent change from baseline will be used for analysis.

#### Other Outcomes

##### Knee extensor voluntary rate of force development (VRFD)

VRFD of the knee extensors will be measured with the subject positioned in the Biodex Dynamometer as described above with the lever arm set to 90° of knee flexion. The subject will extend the knee into the immobilized lever arm with maximal effort over ~500 ms, then immediately relax. The process will be explained to the subject so they understand that they are to produce as much force as possible, as quickly as possible, and then to relax (e.g., pretend you are kicking a tire as hard as you can). The time-series torque signal will be collected at 500 Hz by a Biopac MP150 system (Biopac Systems Inc., Santa Barbara, CA, USA). The trial with the highest VRFD (slope of force tracing between 10 and 90% of maximal force) will be calculated at baseline and post-intervention, and change from baseline will be used for analysis.

##### Twitch-evoked rate of force development (TRFD) and voluntary activation

TRFD and voluntary activation of the knee extensors will be obtained with the subject positioned in the Biodex dynamometer as described above with the lever arm set to at 90° of knee flexion. A surface stimulating electrode will be placed on the distal motor point of the vastus medialis and another on the proximal motor point of the vastus lateralis. Single pulses of incrementally increasing current will be delivered via a Digitimer DS7AH constant current stimulator (Digitimer Ltd., Hertfordshire, UK) until twitch force plateaus. The time-series torque signal will be collected at 500 Hz by a Biopac MP150 system (Biopac Systems Inc., Santa Barbara, CA, USA). TRFD (slope of the twitch response between 10 and 90% of maximal force) at maximal stimulation intensity will be calculated at baseline and post-intervention, and change from baseline will be used for analysis. Subjects will then perform two MVCs separated by 2 min, and during the MVC a doublet will be delivered at maximal intensity (intensity at which twitch force plateaus), followed by a second doublet delivered to resting muscle. Voluntary activation is expressed as [1–(doublet force during MVC/doublet force during relaxation)] ^*^ 100. The time-series torque signal will be collected at 500 Hz by a Biopac MP150 system (Biopac Systems Inc., Santa Barbara, CA, USA). The subject will perform two voluntary activation trials, and the average of the two measures will be recorded at baseline and post-intervention, and change from baseline will be used for analysis.

##### 4-square step test (4SST)

**Figure 2:**
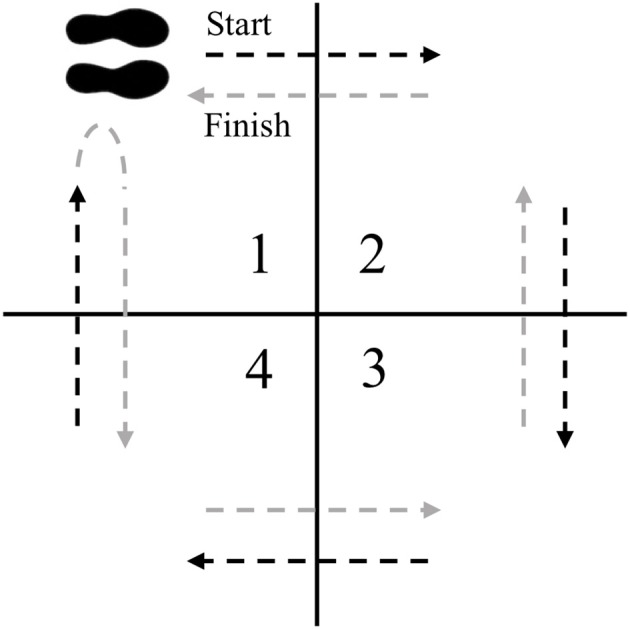
4SST setup. The subject starts in square 1 facing square 2. The subject faces the same direction as they step into squares 2, 3, 4, 1, 4, 3, 2, and then 1.

The 4SST is a test that challenges motor planning and initiation as well as motor sequencing and recall (59, 60). A four-foot by four-foot square will be marked with athletic tape and split into quadrants (Figure 2). The subjects will start in square 1, facing square 2. The subject then steps forward into square 2, laterally to square 3, backwards to square 4, laterally to square 1, laterally to square 4, forwards to square 3, laterally to square 2, and backwards to square 1, facing the same direction throughout the entire sequence. Subjects must place both feet in the specified quadrant before they can move into the next square, and must have at least one foot on the ground at all times. The test will be timed with a stop watch to the nearest 0.01 s. When the subject is ready the investigator will say “ready, set, go.” The timer will begin at “Go” and stopped when the subject has placed both feet back in square 1 after completing a clockwise and counter-clockwise cycle. The subject will perform two trials with 30 s rest between trials, and the fastest will be recorded at baseline and post-intervention. Change from baseline will be used for analysis.

##### Stair climb power

Stair climb power will be calculated as [power = force x velocity] where force = body mass in kilograms x acceleration due to gravity, and velocity = cumulative stair height/stair climb time. The subject will be weighed to the nearest pound, and free weights equivalent to 20% of their body weight will be distributed evenly into two canvas bags. The bags will be set aside for later weighted stair climb testing. The subject will stand at the bottom of a flight of eight stairs (each stair 7″ in height) with feet together. The subject will then be instructed to safely climb the stairs as quickly as possible without skipping any stairs, maintaining at least one foot on a stair at all times. The subject will also be instructed that they may use the handrails if necessary for safety purposes. The test will be timed with a stop watch to the nearest 0.01 s. When the subject is ready the investigator will say “ready, set, go.” The timer will begin at “Go” and stopped when the subject's first foot makes contact with the eighth stair. The subject will perform two trials, and the fastest will be recorded at baseline and post-intervention. Stair climb power will be calculated as described above, and change from baseline will be used for analysis. The subject will repeat the test while carrying the weighted bags equivalent to 20% of their body weight, one bag in each hand. The subject will perform two trials and the fastest will be recorded at baseline and post-intervention. Stair climb power will be calculated as described above, and change from baseline will be used for analysis. 30–60 s rest will be allowed between each trial.

##### Grip strength

Maximal grip strength will be obtained with a Jamar hydraulic grip strength dynamometer (Performance Health, LLC, Akron, OH USA). Handle position of the dynamometer will be standardized at position II (61, 62). Hand dominance will be determined by asking the subject if they were right-handed or left-handed. The dominant hand will be tested first. The subject will be seated with the shoulder in neutral position and the elbow unsupported and flexed to 90° with the forearm and wrist in neutral position (61). The subject will squeeze the dynamometer handle as hard as possible for 3 s. Maximal force will be recorded in kg. The subject will perform three trials with each hand, and 15 s rest will be allowed between trials. Average force of the three trials for each hand will be recorded at baseline and post-intervention, and change from baseline will be used for analysis.

### Exercise Intervention

Each subject will perform their prescribed exercise three times per week for 12 weeks. Time between visits will generally be 48–72 h, but may range from 24 to 96 h to meet the demands of the subject's schedule. Subjects may be allowed to exercise on consecutive days one time per week, but must have at least 48 h before a third exercise session is completed (for example, Monday, Tuesday, Thursday is acceptable, but Monday, Tuesday, Wednesday is not). Prior to each exercise session an exercise supervisor will perform a brief medical safety check. If the subject reports changes in health status that are concerning, has abnormal vital signs (blood pressure readings that exceed 170 mmHg for systolic blood pressure or 100 mmHg for diastolic blood pressure), or has changed medications/dosage, they will not be allowed to continue exercising until the study physician has reviewed the changes and clears the subject. After the medical safety check, the subject will perform a 5-min warm-up on the stationary bike at a low intensity (i.e., output 50% or less of their maximal output during the GXT). After the subject has completed the prescribed exercise for the day they will perform a 5-min cooldown at a low intensity on the stationary bicycle. For the RT and MICT exercise protocols we will employ a pragmatic trial design that follow the recommendations set forth by the ACSM, but that are not necessarily matched by time. There are currently no recommendations for HIIT in older adults, and the exercise sessions are shorter in duration due to the high-intensity nature.

#### Exercise Interventions

##### RT

Subjects will perform 10 resistance exercises for the major muscle groups (Table 4). The training program is lower extremity-focused, with 70% of exercises isolating the lower extremities. For the first 2 weeks subjects will perform 1–2 sets of 15 repetitions for each of the ten exercises, using a weight that elicits a rating of perceived exertion (RPE) at the end of the final repetitions of the respective sets of 5–6 (0–10 scale) as reported by the subject. Rest between sets/exercises will range from 30 to 60 s. Weeks 3–4 subjects will perform 2–3 sets of 12–20 repetitions at an RPE of 5–8 with 60–90 s rest between sets. Weeks 5–8 subjects will perform 3–4 sets of 10–20 repetitions at an RPE of 6–8 with 60–90 s rest between sets. Weeks 9–12 subjects will perform 3–5 sets of 6–20 repetitions at an RPE of 7–8 with 60–90 s rest between sets. Contraction velocity for each exercise will be moderate (180–240°/sec), with duration lasting ~2 s for concentric actions and 2 s for eccentric actions (63). This protocol meets the ACSM resistance training recommendations for older adults (15), and progresses from entry-level to a more demanding protocol. Duration is expected to last ~45–75 min. Subjects will occasionally be asked their perceived effort level on 1–10 scale after individual exercise sets to ensure that they are exercising at the prescribed intensity.

**Table 4:** Resistance training group exercises.

**Daily exercises**	**Rotating exercises**
Leg press Knee extensions Leg curls Calf raises Chest press	Lunges Step-ups (weighted or unweighted) Hip abduction Hip bridge (single- or double-leg) Box squat Sumo squat Planks (knee and elbows or knees and toes) Biceps curls Push-ups (incline or flat) Seated cable pull-down Seated cable row Triceps extensions Shoulder overhead press Lateral arm raises

*The five Daily Exercises are performed during each exercise session. The remaining five exercises each session will be chosen by the exercise supervisor from the list of Rotating Exercises*.

##### MICT

The MICT will be performed on a stationary bicycle (Peloton Interactive, Inc. New York City, NY, USA) interfaced with a computer monitor that plays selected pre-recorded “spin classes.” Heart rate and power output are displayed in real time. The goal of MICT is to maintain an output that elicits the prescribed heart rate throughout the exercise session. Heart rate reserve (HRR) will be calculated by subtracting the subject's resting heart rate (obtained during medical history check at visit 1) from their maximal heart rate (obtained during the GXT). The progression of the 12-week program will go as follows. For the first week subjects will cycle for 20–30 min at 50–60% of their HRR. Week 2 subjects will cycle for 20–30 min at 55–65% of their HRR. Weeks 3–4 subjects will cycle for 30 min at 60–70% of their HRR. Weeks 5–8 subjects will cycle for 30–45 min at 65–75% HRR, and in weeks 9–12 subjects will cycle for 45 min at 70–75% HRR. These ranges are based on recommendations from a meta-analysis describing a dose-response relationship between exercise intensity and VO_2_max adaptations in older adults (64), An exercise supervisor will oversee each exercise session. The target output (in watts) will be determined during the first exercise session by having the subject cycle for 5 min at a cadence of 75 RPMs with a resistance that produces 50 W. Once a consistent heart rate is established (using a chest-strap heart rate monitor), we will increase or decrease the resistance until the target heart rate is maintained. The target output for each subsequent session will be the highest average output from the previous week. If there was no increase from the previous week, target average output will be manually increased by 3%. If the subject is maintaining the target output but not achieving the target heart rate, output will be increased incrementally by 2–5 W until the heart rate is maintained in the target range. During each exercise session the subject will follow the cadence recommendations of the spin class instructor (ranging 50–100 RPMs) while the in-person exercise supervisor modifies the resistance to ensure the output is maintained within the target range. At times the subject may be cycling at the target output but have a heart rate that exceeds the target. In these cases heart rate range takes precedence over output range, and output will be decreased until the target heart rate is maintained. This may occur when cycling at higher cadences (e.g., 90–100 RPMs). At the end of each exercise session subjects will be asked their perceived effort level for the entire session on a 1–10 scale and a Borg 6–20 scale. See Table 5 for exercise duration of each session.

**Table 5:** MICT and HIIT exercise groups cycling duration.

**Session #**	**1**	**2**	**3**	**4**	**5**	**6**	**7**	**8**	**9**	**10**	**11**	**12**
	**Week 1**			**Week 2**			**Week 3**			**Week 4**		
MICT	20	20	30	30	20	30	30	30	30	30	30	30
HIIT	20	20	30	30	20	30	15	15	15	15	15	15
**Session #**	**13**	**14**	**15**	**16**	**17**	**18**	**19**	**29**	**21**	**22**	**23**	**24**
	**Week 1**			**Week 2**			**Week 3**			**Week 4**		
MICT	30	30	30	30	45	30	30	45	30	45	30	45
HIIT	15	20	15	15	20	15	20	15	20	20	15	20
**Session #**	**25**	**26**	**27**	**28**	**29**	**30**	**31**	**32**	**33**	**34**	**35**	**36**
	**Week 1**			**Week 2**			**Week 3**			**Week 4**		
MICT	45	45	45	45	45	45	45	45	45	45	45	45
HIIT	20	20	20	20	30	20	20	30	20	20	30	20

*Duration (in minutes) of cycling for the moderate-intensity continuous training (MICT) and the high-intensity interval training (HIIT) groups for exercise sessions 1–36*.

##### HIIT

Subjects in the HIIT group will use the same stationary bicycle setup as in the MICT group. The progression of the 12-week program will go as follows. Subjects will cycle continuously for 20–30 min at 50–60% of their HRR for the first week, and 20–30 min at 55–65% of HRR for the second week, similar to the MICT group. During the subsequent weeks, subjects will perform bouts of higher intensity cycling (target intensity of 80–100% of their HRR) interspersed with low- intensity rest periods (target intensity of 40–60% of their HRR). Here, the duration of the bout (or interval) will range from as little as 15 s and upwards of 1 min, and rest periods will be matched in a work/rest ratio of 2:1, 1:1, or 1:2 (1:1 on average). During weeks 3–4 the intensity will be at 80–95% of their HRR and the overall duration will last 15–20 min. Weeks 5–8 the intensity will be at 80–100% of their HRR with a duration of 15–30 min. During weeks 9–12 intensity will be at 85–100% of their HRR and duration will last 20–30 min. When the subject begins week three, the target average output will be determined by multiplying the subject's best average output from the first 2 weeks by 1.2. The subject will cycle at the target average output for the first 2–3 min of each session, at 150–300% of their target average output during the high-intensity intervals, and at 25–50% of their target average output during their low-intensity intervals in order to achieve the target heart rate ranges. The target average output for each subsequent week of exercise sessions will be the highest average output from the previous week. If there was no increase from the previous week, target average output will be manually increased by 3%. Subjects will complete 15, 20, or 30-min sessions throughout the study (Table 5) and will begin each session by cycling at the target average output for 2–4 min, followed by several high- and low-intensity intervals. Subjects will cycle at a high intensity for ~6 total min during 15 min sessions, ~8 total min during 20 min sessions, and ~10 total min during 30 min sessions, maintaining an average work/rest ratio of 1:1. The subject will follow the cadence recommendations (ranging 50–100 RPMs) of the spin class instructor while the in-person exercise supervisor modifies the resistance to ensure the output is maintained within the target ranges. It is unlikely that the subject's heart rate will reach 40–60% of HRR during the rest intervals, however, an output that is 25–50% of the target average output should elicit a heart rate that is 40–60% of HRR under normal conditions. Therefore, rest intervals will be long enough that the subject's heart rate will decrease by 5–20 beats per minute prior to the start of the next high-intensity interval. At the end of each exercise session subjects will be asked their perceived effort level for the entire session on a 1–10 scale and a Borg 6–20 scale. See Table 5 for exercise duration of each session.

#### Intervention Discontinuation

Participation in the study will be discontinued if the subject fails to follow the study requirements, experiences serious side effects (e.g., heart attack, exercise related injury), changes the dosage of anti-hypertensive medication or starts taking an exclusionary medication during the course of the study (Table 1), or requests to be removed from the study.

#### Concomitant Exercise and Diet

It is possible that some subjects may be participating in some low-volume physical activity prior to study enrollment and still be eligible for the study (e.g., yoga, yard work). To control for this, all subjects will be encouraged to continue normal activity outside of the study throughout their enrollment in the study. Similarly, study subjects will be asked to maintain their normal diet throughout the study.

#### Adherence

Successful adherence will be defined as a study subject who achieves at least an 80% adherence rate (i.e., attends 29 of 36 exercise sessions).

### Data Management

Data will be entered into the database immediately after each measure is performed by the investigator collecting the measure, and then again at the end of the study by the outcomes assessor. Any incongruous measures will be re-analyzed separately by the outcomes assessor and another blinded investigator. Demographic and intervention group data will be coded and the code will be stored in a locked cabinet unavailable to the outcomes assessor.

### Statistical Methods

The three intervention arms (HIIT, MICT, and RT) will be compared against each other for all primary analyses. For total fat mass and the primary and secondary knee extensor-related outcomes (isokinetic power, muscle volume, MVC, and isokinetic fatigue), we will compute a percentage change from baseline to 12 weeks. For absolute and relative VO_2_max and 6-min walk distance we will compute absolute change from baseline to 12 weeks. We will test differences in group means using one-way ANOVA with Sidak option in SPSS v. 25.0. Tukey *post hoc* tests will be performed if significant differences exist, alpha level set at 0.05. Additionally, as this is a proof-concept, proof of mechanism trial we will calculate effect sizes between groups using the corrected effect size Hedge's g with 95% confidence intervals for small sample sizes (65).

### Harms

In our study, adverse events (AEs) will be defined as an unexpected medical problem that happens during the course of the study related or unrelated to the intervention or assessments. All AEs occurring after informed consent is signed and until study completion will be recorded. An AE that meets the criteria for a serious adverse event (SAE) will be reported to the Ohio University IRB as a SAE. A SAE is defined as an unexpected medical problem that is believed by the investigators to be causally related to the study intervention or assessments and results in any of the following: a life-threatening condition, severe or permanent disability, prolonged hospitalization, or death. Prior to each exercise session the exercise supervisor will perform a brief medical safety check and will ask the subject if they experienced any health issues since the last exercise session. Any issues will be recorded and reviewed by the project manager. The issue will then be recorded as an AE, and will be noted if the project manager believes the issue is related to the intervention (e.g., muscle soreness).

## Anticipated Results

### The Dual-Benefits Hypothesis

The HIIT group will see improvements in endurance measures (VO_2_max, 6-min walk distance, isokinetic fatigue) equal to the MICT group and greater than the RT group, and improvements in muscular characteristics (isokinetic power, MVC, thigh muscle volume, VRFD, TRFD, voluntary activation) equal to the RT group and greater than the MICT group.

### Other Hypotheses

All groups will exhibit similar changes in the 4-square step test and stair climb.

The RT group will see greater improvements in grip strength than either MICT or HIIT groups.

The MICT group will see greater reductions in fat mass than either HIIT or RT groups.

## Discussion

High-intensity interval training has become a popular exercise strategy for young adults for the equivalent or greater health benefits to traditional aerobic training with only a fraction of the time commitment (30). HIIT may also be a highly beneficial exercise strategy for older adults with potential to elicit both muscular and cardiorespiratory adaptations. However, there have been few studies assessing the effects of HIIT in older adults, with the majority being performed in individuals with overt health conditions (33, 35). Although a preliminary study, this is, to our knowledge, the first randomized controlled trial to compare muscular and cardiorespiratory adaptations of bicycle HIIT to adaptations seen in response to traditional bicycle aerobic training or resistance training in generally health older adults. Schjerve et al. (52) compared the effects of HIIT, MICT, and RT in middle-aged, obese adults, reporting large improvements in VO_2_max but no strength gains in the HIIT group. However, this study used a walking protocol for both MICT and HIIT groups, and we postulate the lack of effect on strength was due to the lower levels of muscular activity and force associated with this protocol [e.g., there is evidence that >60% of maximal muscle force must be produced during training to see increases in muscle size and/or function (22)]. HIIT has already been demonstrated to improve cardiorespiratory fitness equal to, or greater than, MICT in older adults (33, 38). Therefore, we propose in this study to determine if HIIT can produce muscular adaptations similar to those seen in response to resistance training. We will be using a cycling protocol for the HIIT group, as improvements in strength and power have been demonstrated in response to HIIT cycling protocols in young adults (28, 30).

The largest potential problem that could arise throughout the course of the study relates to the possibility of AEs. The risk of cardiovascular events in adults participating in HIIT programs is low, as reported by Rognmo et al. (66), who found that of the 4,800+ cardiac rehabilitation patients participating in either HIIT or MICT at a cardiac rehabilitation center, three suffered from cardiac arrest during training (one fatal during MICT, two non-fatal during HIIT). The rate of complications to the number of patient exercise hours was 1 per 23,182 h for HIIT (66). The target population for the current study will include insufficiently active older adults, but those with certain known heart conditions will be excluded from the study, and any subjects who display specific irregular cardiac responses during the baseline GXT (Table 3) will also be excluded. As such, risk of cardiac events is expected to be lower for our population than has been reported in cardiac rehabilitation patients. The American College of Sports Medicine recognizes the risks associated with exercise, but states that the health benefits of exercise outweigh the risks (15).

This study will assess the effectiveness of a high-intensity interval cycling exercise protocol in older adults to produce both cardiorespiratory and muscular adaptations. If shown to be effective it will pave the way for a paradigm shift from volume- to intensity-driven exercise recommendations for older adults, with the ultimate goal of improving health and reducing age-related physical dysfunction.

## Ethics Statement

### Research Ethics Approval

This study has been approved by the Ohio University Institutional Review Board (approval number: 18-F-55).

### Protocol Amendments

Any modifications to the protocol which may impact the conduct of the study, potential benefits to the subject, or affect subjects safety (including changes to study objectives, study design, patient population, sample sizes, study procedures, or significant administrative changes) will require a formal amendment to the protocol. Such amendments will be agreed upon by the principal investigators, and approved by the Ohio University Institutional Review Board prior to implementation.

### Consent

Informed consent will be obtained from each subject after the study has been fully described to the subject, and the consenting investigator believes that the subject fully understands the study requirements and can make an informed decision.

### Confidentiality

Each subject will be assigned a number and experimental data will be recorded using that number. Any identifiable personal information will be kept in a locked cabinet in OMNI, and only the PI will have key access to the information.

### Access to Data

The principal investigators will have direct access to all data sets. Data dispersed to other project team members will be blinded of any identifying subject information.

### Ancillary and Post-trial Care

There are no provisions for ancillary or post-trial care.

### Dissemination Policy

The investigators will provide the subjects with estimates of when the trial will end and when data will be published. The American Heart Association Predoctoral Fellowship requires that all journal articles be made freely available in PubMed Central within 12 months of publication. Eligibility for authorship of manuscripts resulting from this study include (1) substantial contributions to the conception or design of the project, or the acquisition, analysis, or interpretation of the data, AND (2) drafting or revising the manuscript, AND (3) final approval of the manuscript. There is no intention to use professional writers.

## Author Contributions

DT, BC, and DR conceived of the study and initiated the study design. PC and EG helped with implementation. TL provided medical oversight. JS provided statistical expertise. All authors contributed to refinement of the study protocol and approved the final manuscript.

### Conflict of Interest

DT has received research funding from the American Heart Association, the Osteopathic Heritage Foundations, and the John J. Kopchick Fellowship Award from Ohio University. In the past 5-years BC has received research funding from the NIH, Regeneron Pharmaceuticals, Astellas Pharma Global Development, Inc., RTI Health Solutions, Biophytis, and the Osteopathic Heritage Foundations. In the past 5-years BC has received consulting fees from Regeneron Pharmaceuticals, Abbott Laboratories, and the Gerson Lehrman Group. Additionally, BC is co-founder with equity, and serves as the Chief of Aging Research, of AEIOU Scientific, LLC. The remaining authors declare that the research was conducted in the absence of any commercial or financial relationships that could be construed as a potential conflict of interest.
